# Increased Frequency and Distinct Genotypic Patterns of Somatic Aldosterone-Driver Mutations in Aldosterone-Producing Micronodules from Primary Aldosteronism Patients

**DOI:** 10.1007/s12022-026-09912-4

**Published:** 2026-03-20

**Authors:** Jung Soo Lim, Chun-Yi Wu, Zhaoping Qin, Chia-Jen Liu, Desmaré van Rooyen, Dina R. Sapiro, Thomas J. Giordano, Adina F. Turcu, William E. Rainey, Aaron M. Udager

**Affiliations:** 1 Department of Molecular and Integrative Physiology, University of Michigan, Ann Arbor, MI USA; 2 Division of Endocrinology and Metabolism, Department of Internal Medicine, Yonsei University Wonju College of Medicine, Wonju Severance Christian Hospital, Wonju-si, South Korea; 3 Division of Nephrology, Department of Internal Medicine, Taichung Veterans General Hospital, Taichung, Taiwan; 4 Department of Pathology, University of Michigan, Ann Arbor, MI USA; 5 Division of Hematology and Oncology, Department of Internal Medicine, University of Michigan, Ann Arbor, MI USA; 6 Division of Metabolism, Endocrinology, and Diabetes, Department of Internal Medicine, University of Michigan, Ann Arbor, MI USA

**Keywords:** Aldosterone, Primary aldosteronism, Aldosterone-producing adenoma (APA), Aldosterone-producing nodule (APN), Aldosterone-producing micronodule (APM), Aldosterone synthase (CYP11B2), Next-generation sequencing (NGS)

## Abstract

Primary aldosteronism (PA) is the leading cause of endocrine hypertension. With the development of highly specific human aldosterone synthase (CYP11B2) antibodies for immunohistochemistry (IHC), the presence of microscopic subcapsular foci of CYP11B2-postive cells – now termed aldosterone-producing micronodules (APM) – has been documented in normal and PA adrenal glands, however, there continues to be debate regarding the role of APM in the pathogenesis of PA. In this study, CYP11B2 IHC-guided targeted next-generation sequencing (NGS) was utilized to characterize the frequency and spectrum of somatic aldosterone-driver mutations in APM identified within adrenal glands from deceased renal donors and patients with primary aldosteronism (PA). 59 subjects were enrolled (31 deceased renal donors and 28 PA patients), 173 APM were collected using CYP11B2-guided IHC (65 APM from 31 deceased renal donors and 108 APM from 28 PA patients), and NGS data was successfully obtained for 131 APM (51 APM from 31 deceased renal donors and 80 APM from 28 PA patients). Importantly, NGS identified aldosterone-driver mutations in a significantly higher proportion of APM from PA patients relative to deceased renal donors (71.3% compared to 45.1%; P-value < 0.05) – with the vast majority in either group being missense mutations in the L-type calcium channel gene *CACNA1D*. Interestingly, in PA patients, the presence of APA/APN harboring *CACNA1D* or *ATP1A1* mutations was significantly associated with an increased frequency of APM with *CACNA1D* mutations, while APM from adrenal glands with KCNJ5 mutation-bearing APA/APN were more likely to be mutation-negative (*P*-values < 0.001 and < 0.05, respectively). Finally, clinical outcomes for PA patients were significantly associated with APM genotype, with post-surgical cure showing higher frequencies of *KCNJ5*-mutant and mutation-negative APM and a lower frequency of *CACNA1D*-mutant APM. Overall, our data support a link between the acquisition of somatic aldosterone-driver mutations in APM and PA pathogenesis and suggest the possibility of diverse genetic mechanisms underlying APM development in normal and PA adrenal glands. Furthermore, these results suggest that molecular diagnostic testing of CYP11B2-positive adrenal cortical lesions may help risk stratify patients in clinical practice.

**Supplementary Information:**

The online version contains supplementary material available at 10.1007/s12022-026-09912-4.

## Introduction

Primary aldosteronism (PA) results from renin-independent / dysregulated aldosterone production by one or both adrenal glands. PA represents the most common form of secondary hypertension, accounting for 6–16% of all hypertensive patients and between 15 and 25% of patients with resistant hypertension [1–3]. Chronic inappropriately elevated aldosterone levels lead to target tissue damage and remodeling, resulting in higher cardiovascular risk compared to patients with essential hypertension [4–6]. Based on the source(s) of aldosterone excess, PA causes can be generally categorized into unilateral (UPA) and bilateral (BPA) forms [7]. Most UPA results from a single dominant CYP11B2 (aldosterone synthase)-positive aldosterone-producing adenoma (APA) or a smaller (< 10 mm) aldosterone-producing nodule (APN) [8]. For patients with solitary APA or APN, PA can usually be cured by unilateral adrenalectomy. Conversely, BPA is caused by inappropriate aldosterone production from both adrenal glands, which is generally treated with medications, including mineralocorticoid receptor antagonists. Distinguishing UPA from BPA is primarily done using adrenal vein sampling (AVS), which assesses relative aldosterone production from both adrenal glands and may allow lateralization of the source of excess aldosterone. However, recent studies have shown that some patients with apparent UPA (i.e., lateralized PA on AVS) do not achieve biochemical cure of PA following surgery [9], suggesting the need for careful adrenal histopathologic assessment and long-term post-surgical follow-up. Indeed, these patients often exhibit multiple CYP11B2-positive adrenal cortical lesions in the excised adrenal gland, and the lack of PA cure suggests asymmetric bilateral PA (APBA) despite AVS lateralization [10].

Recent studies suggest that BPA and ABPA have a unique histopathologic adrenal presentation with the presence of multifocal CYP11B2-expressing cell clusters [10, 11]. These have been termed aldosterone-producing cell clusters (APCC) [12], as well as CYP11B2-positive cell foci or megafoci [13]. Recently, an international histopathology consensus established a nomenclature (HISTALDO) for the histopathologic features of adrenal glands from PA patients based on CYP11B2 immunohistochemistry (IHC) [8]. These recommendations were subsequently adopted by the World Health Organization Classification of Tumors of Endocrine Organs [14]. As part of the HISTALDO nomenclature, CYP11B2-positive APCC have been renamed aldosterone-producing micronodules (APM), and thus, the spectrum of CYP11B2-positive lesions in adrenal glands from PA patients includes APA, APN, and APM. Importantly, we and others have shown that somatic mutations are a common feature in each of these categories [11, 15–19].

The application of whole-exome next-generation sequencing (NGS) to PA research has led to the discovery of a series of recurrent somatic mutations in UPA and BPA adrenal glands that are thought to cause inappropriate aldosterone production (i.e., “aldosterone-driver mutations”) [4, 20, 21]. Potassium inwardly rectifying channel subfamily J member 5 (*KCNJ5*) is the most common gene with somatic mutations in UPA. *KCNJ5* variants cause abnormal sodium permeability and activate voltage-dependent calcium channels, thereby leading to aldosterone overproduction [22]. Activating mutations in calcium voltage-gated channel subunit alpha1 D (*CACNA1D*) cause an increase in calcium influx and aldosterone production and appear to be the most common variants observed in BPA and in lateralized PA cases without biochemical cure following unilateral adrenalectomy [10, 11, 23, 24]. Less common somatic aldosterone-driver mutations have been identified in the following genes: ATPase Na+/K+ transporting subunit alpha 1 (*ATP1A1*) [23]; ATPase plasma membrane Ca2 + transporting 3 (*ATP2B3*) [25]; catenin beta 1 (*CTNNB1*) with G protein subunit alpha 11 (*GNA11*) or G protein subunit alpha q (*GNAQ*) [26]; chloride voltage-gated channel 2 (*CLCN2*) [27, 28]; calcium voltage-gated channel subunit alpha1 H (*CACNA1H)* [29]; cell adhesion molecule 1 (*CADM1*) [30]; and, solute carrier family 30 member 1 (*SLC30A1*) [31]. Importantly, CYP11B2 IHC-guided tissue capture followed by deep targeted NGS suggests that greater than 95% of APA harbor known aldosterone-driver mutations [32].

The challenging nature of collecting sufficient DNA from the much smaller APM has limited genetic studies of these lesions. The current study was undertaken to characterize the frequency and spectrum of aldosterone-driver mutations in APM from PA patient adrenal glands and “normal” adrenal glands obtained from deceased renal donors.

## Materials and Methods

### Human Adrenal Gland Samples

All experimental procedures carried out in this study were reviewed and approved by the Institutional Review Board of the University of Michigan (HUM00106809 and HUM00083056). A waiver of informed consent was granted for the use of archival clinical adrenal specimens. Adrenal glands obtained from deceased renal transplantation donors at the University of Michigan were processed into formalin-fixed paraffin-embedded (FFPE) tissue blocks. While the hypertensive and PA status of these donors is not known, no gross abnormalities were detected on routine pathologic examination of the adrenal gland, and no adrenal cortical nodules were identified on microscopic examination of hematoxylin and eosin (H&E) and corresponding CYP11B2 IHC slides. Clinical FFPE adrenal gland tissue blocks were obtained from PA patients who underwent standard of care unilateral adrenalectomy at University of Michigan. Post-surgical clinical outcomes were defined as follows: (1) cure = normalization of serum potassium and blood pressure without antihypertensive medications; and, (2) improvement = normalization of serum potassium and blood pressure with reduced number of antihypertensive medications.

### Identification and Classification of Aldosterone-Producing Adrenal Cortical Lesions

Aldosterone-producing adrenal cortical lesions were identified in sectioned adrenal gland tissue using H&E-stained slides and CYP11B2 IHC, as described previously [33], and classified using the HISTALDO framework with similar criteria [8]. Briefly, APA were classified as CYP11B2-positive nodular adrenal cortical lesions greater than or equal to 10 mm in maximum size, while APN were similar such lesions less than 10 mm in maximum size. APM were classified as microscopic subcapsular CYP11B2-positive adrenal cortical lesions that were difficult to distinguish from the adjacent adrenal cortex on corresponding H&E slides and lacked distinctly nodular CYP11B2 staining. All CYP11B2 IHC analyses were carried out via manual review of glass slides with a microscope or scanned whole-slide images. CYP11B2-positive lesions with questionable HISTALDO classification were reviewed together by two study team members with extensive experience (W.E.R and A.M.U.) to achieve consensus.

### CYP11B2 IHC-Guided Tissue Capture and DNA Extraction

Using the corresponding CYP11B2 IHC slide as a guide, APM tissue was manually macrodissected from multiple adjacent unstained FFPE tissue sections using a scalpel, as described previously [32]. DNA was extracted from macrodissected APM tissue samples using the AllPrep FFPE DNA/RNA Kit (Qiagen, Venlo, Netherlands) and quantitated using a Qubit 3.0 Fluorometer (Thermo Fisher Scientific, Waltham, MA).

### Targeted NGS of Aldosterone-Driver Mutations

Targeted NGS libraries were generated from up to 20 ng of FFPE-extracted DNA using two custom Ion AmpliSeq NGS panels (Thermo Fisher Scientific). As described previously, the APCCv4 panel targets the complete coding regions of the following known aldosterone-driver genes: *ATP1A1*, *ATP2B3*, *CACNA1D*, *CACNA1H*, *CADM1*, *CLCN2*, *CTNNB1*, *KCNJ5*, *GNA11*, and *SLC30A1* [30, 31, 34]. Briefly, barcoded APCCv4 NGS libraries were constructed using the Ion AmpliSeq Library Kit Plus (Thermo Fisher Scientific) and pooled for sequencing on an Ion GeneStudio S5 Prime System (Thermo Fisher Scientific) with an Ion Chef Instrument (Thermo Fisher Scientific). APCCv4 NGS reads were demultiplexed and aligned to the human genome (hg19) using Ion Torrent Suite software (Thermo Fisher Scientific) prior to quality control (GC) assessment using the coverageAnalysis plugin. Variants were identified from sequenced APCCv4 NGS libraries that passed standard QC metrics (mean depth > 500X and uniformity > 70%) using the variantCaller plugin and annotated using validated in-house bioinformatics pipelines prior to manual curation by an experienced molecular pathologist (A.M.U.). The APCCv5 panel is a novel Ion AmpliSeq HD panel that targets the mutational hotspot regions of the following known aldosterone-driver genes: *ATP1A1*, *ATP2B3*, *CACNA1D*, *CACNA1H*, *CLCN2*, *CTNNB1*, *KCNJ5*, *GNA11*, and *SLC30A1*. Briefly, barcoded APCCv5 NGS libraries were constructed from Uracil-DNA Glycosylase (UDG)-treated FFPE-extracted DNA using the Ion AmpliSeq HD Library Kit (Thermo Fisher Scientific) prior to being pooled for sequencing, demultiplexed, and aligned to hg19, as described above. QC assessment of sequenced APCCv5 NGS libraries was performed using the molecularCoverageAnalysis plugin in Ion Torrent Suite (Thermo Fisher Scientific), and variants were identified from libraries that passed standard QC metrics (median functional molecules > 100 and molecular uniformity > 70%) using the variantCaller plugin prior to annotation and manual curation, as described above. All samples without prioritized somatic variants on APCCv5 were re-sequenced using APCCv4 to identify potential somatic variants outside of the targeted mutation hotspot regions.

### Statistical Approaches

All statistical analyses were performed in R using standard approaches as applicable, including: Fisher’s exact test (with or without Holm-adjusted post-hoc pairwise comparisons); chi-squared test with Yates correction; Pearson correlation coefficient; Student’s t-test; Mann-Whitney U-test; and, Kruskal-Wallis test.

## Results

### Cohort Characteristics

Adrenal glands from 31 deceased renal donors and 28 patients with PA were included in the study cohort (Supplemental Tables 1 and 2). While there was a significantly higher proportion of White deceased renal donors compared to PA patients (*P*-value = 0.033), no significant sex or age differences between the two populations were present. Post-surgical clinical follow-up information was available for 25 of the PA patients: 7 experienced post-surgical cure, while 18 had post-surgical improvement only (see Materials and Methods for details). Importantly, while post-surgical cure was significantly associated with younger age and female sex (*P*-value < 0.01), there was no significant association between post-surgical outcome and race, type of adrenal nodule (i.e., APA vs. APN), or APA/APN genotype – although given its relatively small size, the PA cohort may be underpowered to detect some factors associated with clinical outcome.

**Fig. 1:**
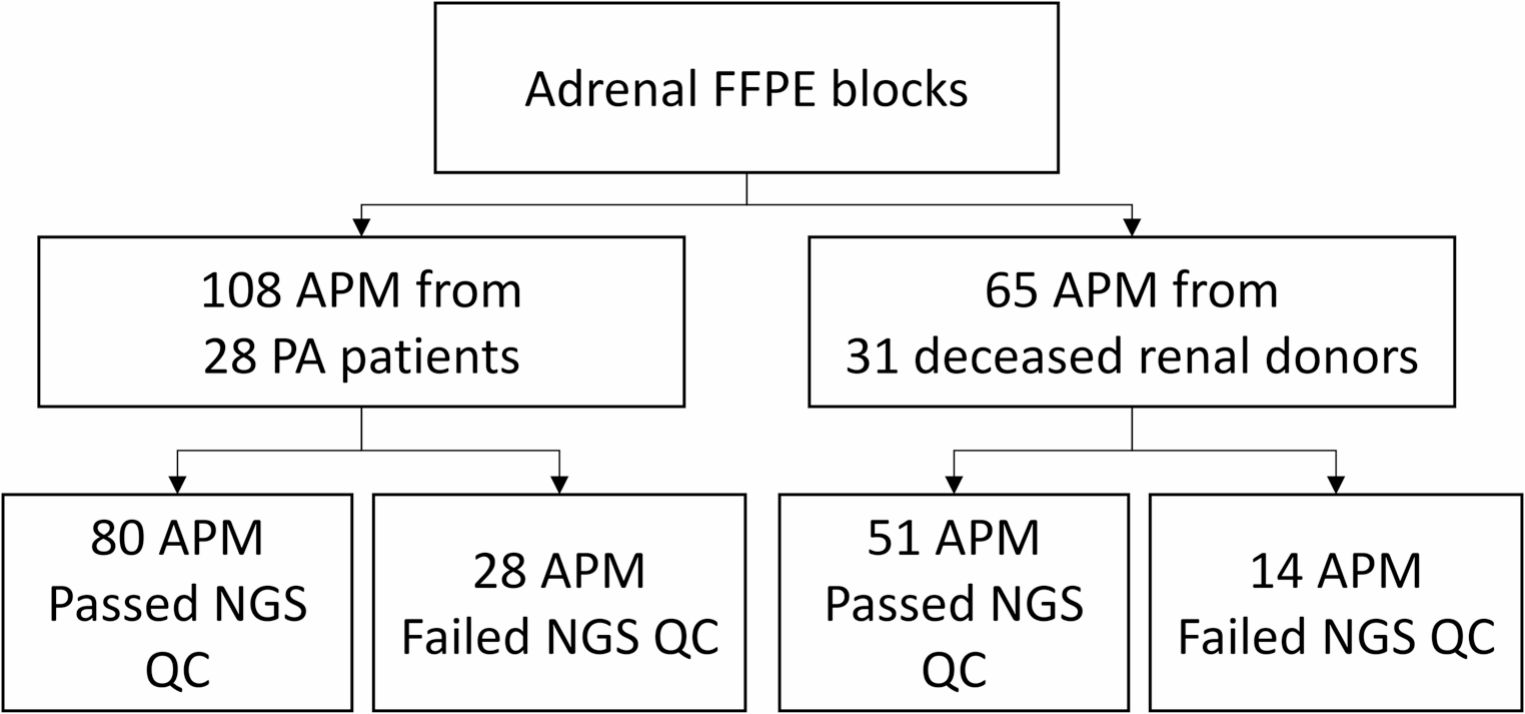
Study workflow diagram. Somatic aldosterone-driver mutations in aldosterone-producing micronodules (APM) were analyzed using formalin-fixed paraffin-embedded (FFPE) adrenal glands from deceased renal donors and patients with primary aldosteronism (PA). Immunohistochemistry for CYP11B2 (aldosterone synthase) was utilized to localize APM, which then underwent tissue capture from serial sections, DNA extraction, and targeted next-generation sequencing (NGS) to identify somatic aldosterone-driver mutations. The number of APM that were successfully analyzed by NGS or failed NGS quality control are indicated.

**Fig. 2:**
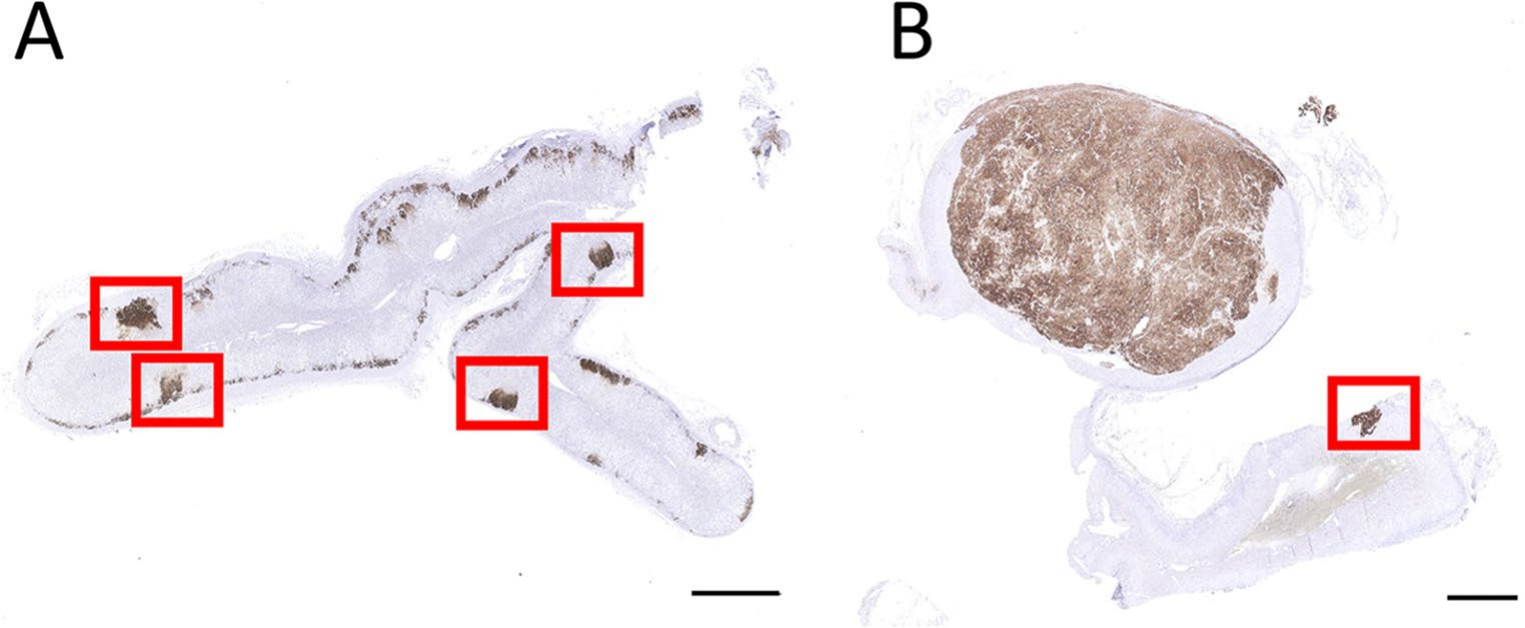
CYP11B2 immunohistochemistry highlights aldosterone-producing micronodules (APM) in adrenal gland tissue. Representative photomicrographs of CYP11B2 immunochemistry (IHC) stains in sectioned adrenal gland tissue from a deceased renal donor (**A**) or PA patient (**B**). CYP11B2 IHC highlights the presence of multiple APM in the deceased renal donor (without a concurrent APA or APN) and a concurrent APA and APM in the PA patient. APM are shown in red boxes. Scale bar = 4 mm

CYP11B2 IHC performed on FFPE adrenal gland tissue identified 65 APM for analysis in 31 deceased renal donors and 108 APM for analysis in 28 PA patients (Fig. 1). While there was a significantly increased number of APM per adrenal gland in PA patients compared to deceased renal donors (range = 1–17 compared to 1–7; *P*-value = 0.036), no significant age, sex, or race associations with APM number were detected. CYP11B2 IHC also highlighted at least one concurrent APA or APN in all adrenal glands from PA patients, while no APA or APN were identified in adrenal glands from deceased renal donors. Figure 2 displays representative CYP11B2 IHC staining in adrenal gland tissue, illustrating the presence of multiple APM in a deceased renal donor (without a concurrent APA or APN) and concurrent APA and APM in a PA patient.

### Aldosterone-Driver Mutations are More Common in APM from PA Patients than Deceased Renal Donors

**Fig. 3:**
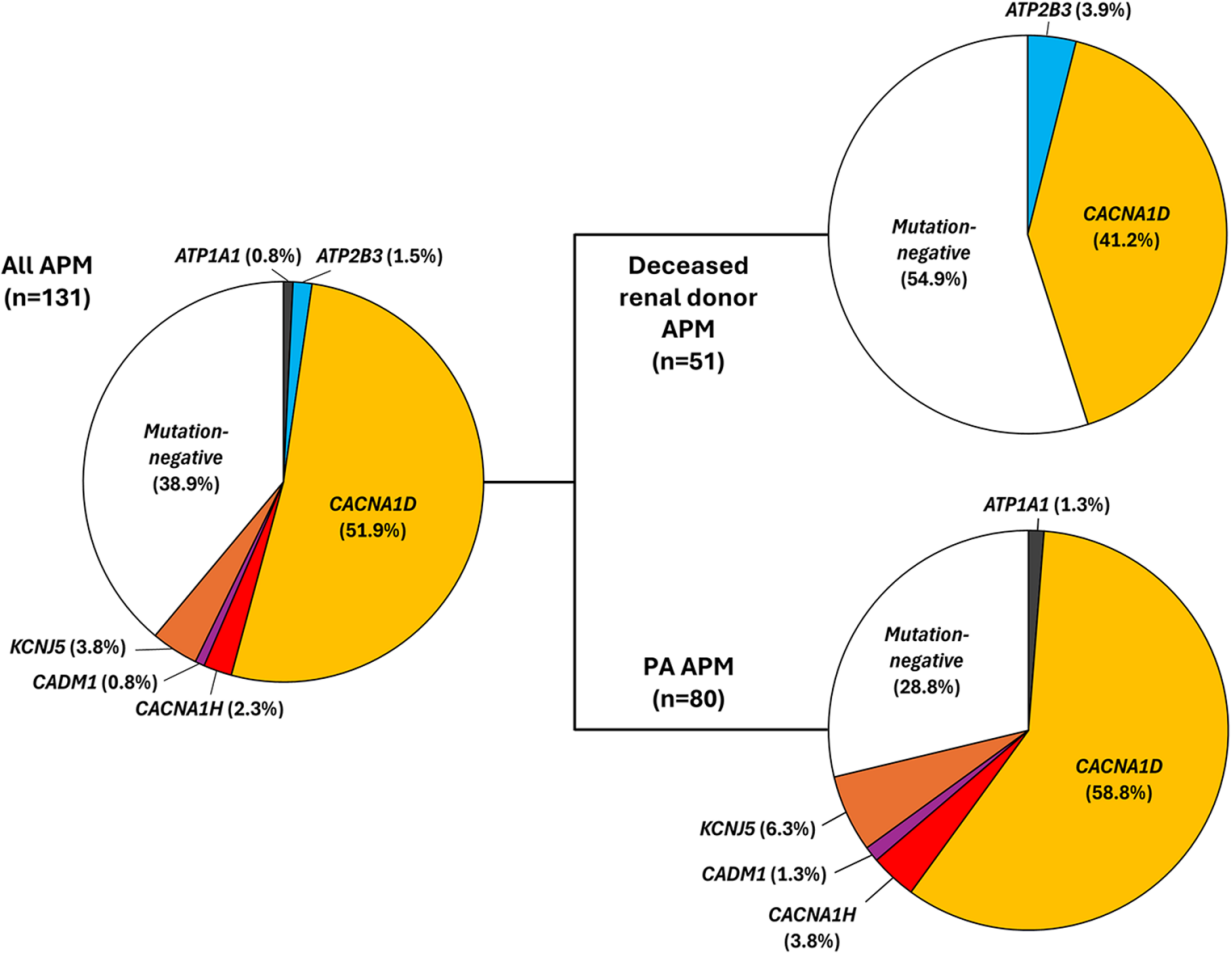
Somatic aldosterone-driver mutations are more prevalent in aldosterone-producing micronodules (APM) from primary aldosteronism (PA) patients than deceased renal donors. Pie charts depicting the overall frequency and distribution of somatic aldosterone-driver mutations in all APM analyzed (left) and then subdivided by subject status (right; i.e., deceased renal donor vs. PA patient). While CACNA1D mutations are the most common aldosterone-driver mutation in both groups, the prevalence of aldosterone-driver mutations is significantly higher in PA patients (*P*-value < 0.05)

After CYP11B2 IHC-guided tissue capture, targeted NGS using custom aldosterone-driver mutation panels was performed on all APM from our cohort; successful NGS results were generated for 51 APM from 31 deceased renal donors and 80 APM from 28 PA patients (Fig. 1 and Supplemental Tables 1 and 2), and there was no significant difference in the proportion of APM samples that failed NGS between the two populations. Overall, somatic aldosterone-driver mutations were identified in a majority of APM analyzed (*n* = 80; 61.1%) with *CACNA1D* mutations being the common APM genotype (*n* = 68; 85.0%). Interestingly, a significantly higher proportion of APM from PA patients harbored an aldosterone-driver mutation compared to deceased renal donors (71.3% compared to 41.2%; *P*-value = 0.003); however, no significant difference was detected in the distribution of APM genotypes among these populations (Fig. 3). For deceased renal donors, mutations were identified in *CACNA1D* (*n* = 21; 41.2%) and *ATP2B3* (*n* = 2; 3.9%) with the remainder negative for known mutations (*n* = 28; 54.9%), while for PA patients, mutations were detected in *CACNA1D* (*n* = 47; 58.6%), *KCNJ5* (*n* = 5; 6.3%), *CACNA1H* (*n* = 3; 3.8%), *ATP1A1* (*n* = 1; 1.3%), and *CADM1* (*n* = 1; 1.3%) with the rest negative for known mutations (*n* = 23; 28.8%). The full list of APM aldosterone-driver mutations identified in this study is available in Table 1.

**Table 1:** Summary of somatic aldosterone-producing micronodules (APM) aldosterone-driver mutations identified in this study

Gene	Somatic mutation	PA adrenal APM	Deceased renal donor adrenal APM
*ATP1A1*	c.995T > G (p.V332G)	1	0
*ATP2B3*	c.1248_1258del (p.K416_F418delinsN)	0	1
	c.1270_1275del (p.L425_V426del)	0	1
CACNA1D*	c.815T > C (p.L272P)	1	0
	c.827T > C (p.L276P)	0	1
	c.827T > G (p.L276R)	1	0
	c.1207G > C [p.G403R (exon8A)]	4	2
	c.1207G > C [p.G403R (exon8B)]**	3	0
	c.1904 C > T (p.S635F)	0	1
	c.1949T > A (p.I650N)	1	0
	c.1955 C > T (p.S652L)	1	0
	c.1957 C > A (p.L653M)	1	0
	c.1958T > A (p.L653Q)	0	1
	c.2234 A > C (p.N745T)	1	0
	c.2239T > C (p.F747L)	1	0
	c.2239T > G (p.F747V)	11	3
	c.2241 C > A (p.F747L)	1	0
	c.2248 A > G (p.I750V)	0	1
	c.2250 C > G (p.I750M)	3	0
	c.2942 A > C (p.K981T)	1	1
	c.2943G > T (p.K981N)	0	1
	c.2969G > A (p.R990H)	3	0
	c.2978G > C (p.R993T)	0	1
	c.2978G > T (p.R993M)	1	0
	c.2992_2993delinsAT (p.A998I)	1	0
	c.2993 C > T (p.A998V)	2	0
	c.3019T > C (p.C1007R)	0	1
	c.3044T > G (p.I1015S)	0	1
	c.3374G > A (p.R1125H)	0	1
	c.3409 A > T (p.I1137F)	1	0
	c.3446G > T (p.G1149V)	0	1
	c.3451G > T (p.V1151F)	1	0
	c.3452T > C (p.V1151A)	1	0
	c.3458T > A (p.V1153D)	1	0
	c.3458T > G (p.V1153G)	0	1
	c.3744 C > A (p.F1248L)	1	0
	c.4007 C > G (p.P1336R)	2	1
	c.4012G > A (p.V1338M)	2	3
	c.4342 A > T (p.I1448F)	1	0
*CACNA1H*	c.4289T > C (p.I1430T)	3	0
*CADM1*	c.1124_1133delinsA (p.A375_G378delinsD)	1	0
*KCNJ5*	c.433G > C (p.E145Q)	1	0
	c.440_442del (p.E147_T148delinsA)	1	0
	c.461T > G (p.F154C)	1	0
	c.472 A > G (p.T158A)	1	0
	c.503T > G (p.L168R)	1	0

*Amino acid changes based on NM_00128839 (unless otherwise noted)

**Amino acid change based on NM_000720

### Distinct APM Aldosterone-Driver Genotypes in Subsets of PA Patients

**Fig. 4:**
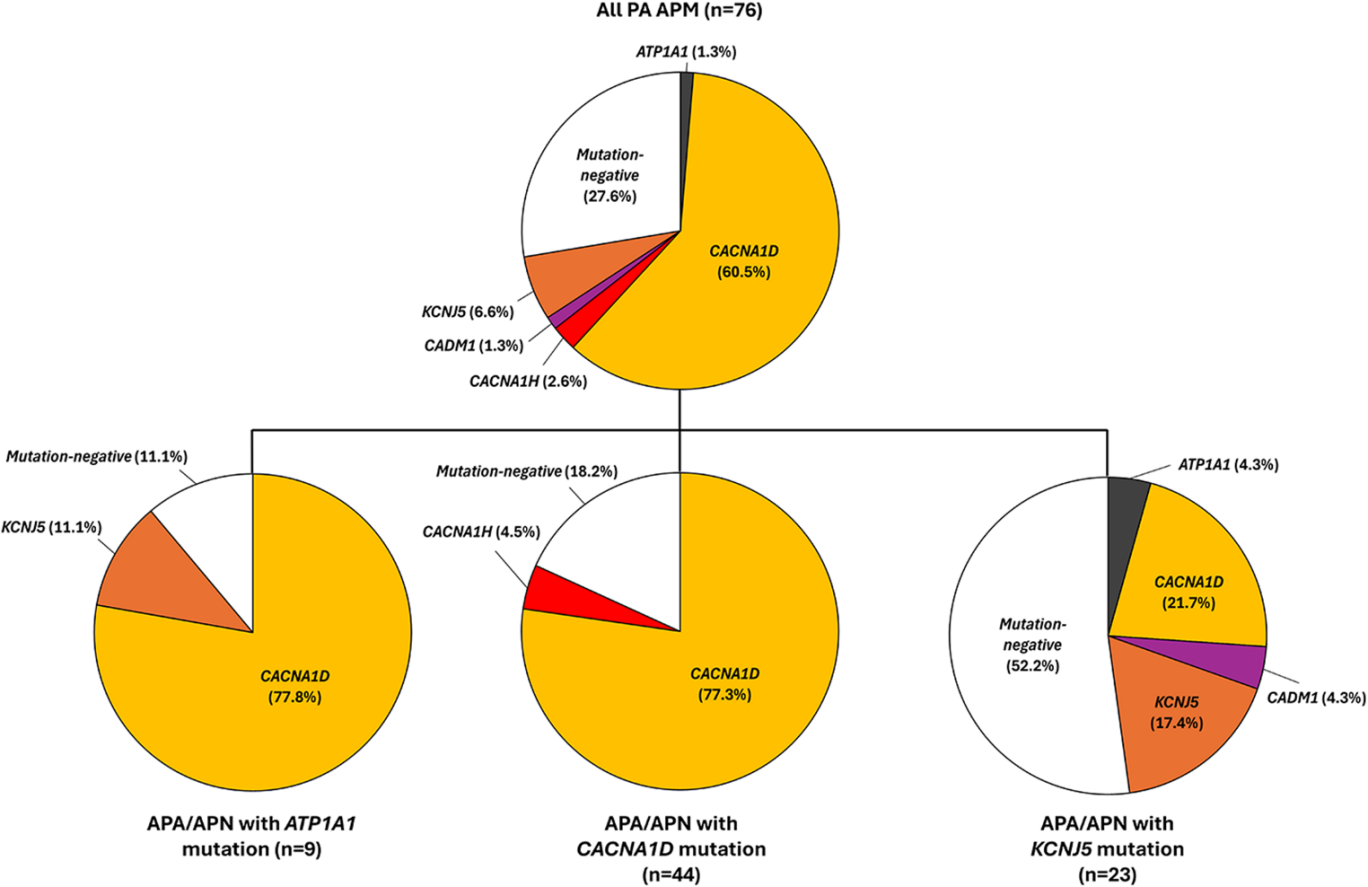
The pattern of somatic aldosterone-driver mutations in aldosterone-producing micronodules (APM) varies across genetic subtypes of primary aldosteronism (PA) patients. Pie charts depicting the overall frequency and distribution of somatic aldosterone-driver mutations in all APM analyzed from PA patients (top) and then subdivided by genotype of concurrent aldosterone-producing adenoma (APA) and/or aldosterone-producing nodule (APN) (bottom; i.e., ATP1A1, CACNA1D, and KCNJ5). While the vast majority of APM in PA patients with ATP1A1 or CACNA1D APA/APN harbored CACNA1D mutations, these mutations were present in less than a quarter of APM in PA patients with KNCJ5 APA/APN (*P*-value < 0.001)

As described above, at least one concurrent APA or APN was identified by CYP11B2 IHC in all PA patients, including 10 with an APA, 14 with one APN, and 4 with two APN. Patient-level APA/APN aldosterone-driver genotypes included: *CACNA1D* APA/APN (*n* = 10); *KCNJ5* APA/APN (*n* = 8); *ATP1A1* APA/APN (*n* = 5); two *CACNA1D* APN (*n* = 3); *ATP2B3* APA (*n* = 1); and, one *KNCJ5* APN and one *ATP1A1* APN (*n* = 1). The full list of APA/APN aldosterone-driver mutations is available in Supplementary Table 2. While no significant differences were detected between the APA/APN genotype and sex, age, race, or APM number, the frequency of specific APM aldosterone-driver mutations varied significantly across PA patients with *CACNA1D*, *KCNJ5*, and *ATP1A1* mutations (Fig. 4). While *CACNA1D* mutations comprised a substantial majority of APM in PA patients with *CACNA1D-* or *ATP1A1*-bearing APA/APN (77.3% and 77.8%, respectively), only a minor subset of APM in PA patients with *KCNJ5*-bearing APA/APN harbored *CACNA1D* mutations (21.7%; *P*-values < 0.001 and < 0.05, respectively). In contrast, while over half of APM in PA patients with *KCNJ5*-bearing APA/APN were negative for known aldosterone-driver mutations, less than 20% of APM in PA patients with *CACNA1D*-bearing APA/APN were mutation-negative (*P*-value < 0.05). 80% of the APM with *KCNJ5* mutations (*n* = 4) occurred in PA patients with *KCNJ5*-bearing APA/APN (*P*-value < 0.05), however, sample size limited post-hoc pairwise comparisons among specific APA/APN genotypes. Importantly, the *KCNJ5* genotypes detected were discordant between APA/APN and APM – excluding the possibility of intra-adrenal sample contamination or *KCNJ5* germline mosaicism. Finally, when APM from PA patients were stratified by clinical outcome, there was a significant difference in the distribution of APM genotypes among PA patients with post-surgical cure compared to those with post-surgical improvement only (*P*-value < 0.005); PA patients with post-surgical cure showed relatively more frequent *KCNJ5*-mutant APM (16.7% vs. 2.1%) and mutation-negative APM (41.7% vs. 23.4%) and relatively less frequent *CACNA1D*-mutant APM (33.3% vs. 70.2%).

## Discussion

Over the past 15 years, research from multiple groups has demonstrated that sporadic (non-familial) PA frequently results from the acquisition of somatic aldosterone-driver mutations in genes encoding ion channels and transporters. Directly or indirectly, these mutations cause increased intracellular calcium leading to renin-independent CYP11B2 expression and excess aldosterone production. HISTALDO, the recent international consensus for the pathologic evaluation of PA adrenal glands, utilizes a combination of histomorphology and CYP11B2 IHC to categorize aldosterone-producing adrenal cortical lesions into APA, APN, and APM [8]. The smallest of these lesions, APM are thought to represent the earliest stages of aldosterone dysregulation. However, it remains unclear how APM are involved in the development of larger aldosterone-producing lesions (APA or APN) and/or the clinical phenotypes of sporadic PA (UPA, BPA, ABPA, etc.). To obtain more insight into the origins of PA, this study aimed to examine the frequency and distribution of somatic aldosterone-driver mutations in APM from PA patient adrenal glands and “normal” adrenal glands obtained from deceased renal donors.

To date, somatic mutations in eleven genes have been identified in APA and APN [22, 23, 25–31]. These genes encode ion channels (*KCNJ5*, *CACNA1D*, *CACNA1H*, and *CLCN2*), ion transporters (*ATP1A1*, *ATP2B3*, and *SLC30A1*), or cell signaling proteins (*CTNNB1*, *GNAQ*, *GNA11*, and *CADM1*). Herein, we demonstrated that mutations in six of these genes also occur in APM obtained from PA patients or deceased renal donors. Similar to APA and APN, the vast majority of these aldosterone-driver mutations (91.3%) occurred in just two ion channel genes (*KCNJ5* and *CACNA1D*), and together, mutations in these genes were present in more than half of all studied APM (55.7%). Importantly, *CACNA1D* mutations were the main aldosterone-driver mutation in APM from both PA patients or deceased renal donors, and multiple APM within the same adrenal gland typically harbored unique mutations. These findings are similar to those previously reported for normal adrenal glands, as well as those obtained from patients with BPA or “imaging-negative” UPA (i.e., lateralized PA without a radiographic apparent adrenal lesion) [11, 15]. According to in vitro adrenal cell-based studies, mutated *CACNA1D* increases aldosterone production, which is significantly reduced by the calcium channel blocker nifedipine [35]. However, how APM with *CACNA1D* mutations affect the continuum of autonomous aldosteronism progressing to PA remains to be determined. Additional studies are needed to define the mechanisms leading to the high rate of APM aldosterone-driver mutations in the *CACNA1D* gene and further confirm their potential as therapeutic targets for BPA/ABPA.

Two pathogenic models of APA development have been proposed [20, 36]. One postulates that the presence of somatic mutations in various aldosterone-driver genes leads to the occurrence of APM, which can develop into APA through the formation of CYP11B2-positive micronodular lesions [termed possible APCC-to-APA translational lesions (pAATL)]. The other is a two-hit model: abnormal cell proliferation in the ZG caused by additional genetic and/or environmental factors and acquisition of aldosterone-driver mutations induces APA formation. Our prior data – as well as the current study – have shown that APM typically harbor aldosterone-driver mutations that are also found in APA (i.e., *CACNA1D*, etc.), supporting the hypothesis that APM can be a precursor for APA [11, 15, 37]. Nevertheless, the mechanisms of cell proliferation and/or nodulation in PA adrenal cortical lesions are not fully understood.

This is the first study with a sufficient number of APM genotypes to be able to assess the impact of concurrent APA/APN genotype and/or clinical outcome on the distribution of aldosterone-driver mutations. Interestingly, we observed striking differences among the three most common APA/APN aldosterone-driver genotypes: *ATP1A1*, *CACNA1D*, and *KCNJ5*. While the spectrum of APM aldosterone-driver mutations was very similar for PA patients with APA/APN harboring *ATP1A1* or *CACNA1D* mutations – with *CACNA1D* being the dominant genotype (nearly 80% of APM) – a majority of APM in PA patients with *KCNJ5*-mutant APA/APN were mutation-negative, and less than 25% harbored a *CACNA1D* mutation; in addition, four (80%) of the APM with *KCNJ5* mutations occurred in PA adrenals with *KCNJ5*-mutant APA/APN. While we did not detect a statistically significant association between *KCNJ5*-mutant APA/APN and clinical outcome in our PA cohort (possibly due to the small sample size), young age and female sex – which are associated with somatic *KCNJ5* APA/APN mutations in PA patients [19] – were significantly associated with post-surgical cure. Interestingly, the distribution of APM genotypes was also related to clinical outcome, as post-surgical cure was correlated with increased frequency of *KNCJ5*-mutant and mutation-negative APM and decreased frequency of *CACNA1D*-mutant APM. Overall, these data suggest that the cellular and molecular environment of *KCNJ5*-bearing PA adrenals may be very different from other APA/APN genotypes, and given the association between UPA and *KCNJ5*-mutant APA/APN, these findings indicate the potential for distinct genetic mechanisms underlying the development of UPA and BPA/ABPA. Future additional studies are needed to confirm and extend these observations.

Limitations of the present study include the inability to determine the aldosterone-driver mutation status in almost one-quarter of studied APM. While the limited amount of DNA that can be captured from APM – as well as DNA degradation and fragmentation associated with FFPE tissue – makes comprehensive NGS analyses challenging, our targeted NGS approaches were able to successfully determine the APM genotype for the vast majority of samples. Still, it is difficult to successfully carry out whole-exome sequencing of APM in order to identify potentially novel aldosterone-driver mutations in these lesions. Improvements in sequencing technology will hopefully address this issue in the coming years. The deceased renal donor adrenals used in this study may also impact on the findings, as these cases were not explicitly screened for PA, and thus, the presence of PA in this cohort cannot be unequivocally excluded. Importantly, however, in all cases, the donor adrenal glands were grossly normal without adrenal cortical nodules on routine microscopic examination and CYP11B2 IHC. Furthermore, given the rarity of PA in the general population, the likelihood of a significant number of randomly selected deceased renal donors with undiagnosed PA is extremely low. Regardless, the current results are foundational by establishing the aldosterone-driver mutation status for the largest set of APM studied to date. In addition, this study provides the methods to localize aldosterone-producing cells, isolate DNA, and define somatic mutations using FFPE tissue – providing a framework for others to use archival adrenal glands for similar studies.

In conclusion, our results support the hypothesis that APM represent the earliest stage of aldosterone dysregulation in PA, and given the very high frequency of *CACNA1D* mutations in these lesions, our findings suggest a central role for calcium channel blockers in clinical management of BPA and ABPA patients. Intriguingly, the observation of a significantly increased proportion of mutation-negative APM from adrenal glands with *KCNJ5* mutation-bearing APA/APN – frequently corresponding to patients with UPA – suggests the possibility of distinct underlying genetic mechanisms for the development of UPA and BPA/ABPA. Furthermore, given the observed associations between young age, female sex, and APM genotypes and post-surgical cure in PA patients, our data suggest that molecular diagnostic testing of CYP11B2-positive adrenal cortical lesions may help risk stratify patients in clinical practice.

## Supplementary Information

Below is the link to the electronic supplementary material.

Supplementary Material 1

Supplementary Material 2

## Data Availability

Data supporting the findings of this study are available within the manuscript and its supplementary information. Next-generation sequencing data is available upon request to qualified investigators.
